# Comparative Profiling of TG2 and Its Effectors in Human Relapsing Remitting and Progressive Multiple Sclerosis

**DOI:** 10.3390/biomedicines10061241

**Published:** 2022-05-26

**Authors:** Damien D. Pearse, Andrew B. Hefley, Alejo A. Morales, Mousumi Ghosh

**Affiliations:** 1 The Miami Project to Cure Paralysis, University of Miami Miller School of Medicine, Miami, FL 33136, USA; dpearse@med.miami.edu (D.D.P.); abh87@miami.edu (A.B.H.); amorales1@med.miami.edu (A.A.M.); 2 The Department of Neurological Surgery, University of Miami Miller School of Medicine, Miami, FL 33136, USA; 3 The Neuroscience Program, University of Miami Miller School of Medicine, Miami, FL 33136, USA; 4 The Interdisciplinary Stem Cell Institute, University of Miami Miller School of Medicine, Miami, FL 33136, USA; 5 Department of Veterans Affairs, Veterans Affairs Medical Center, Miami, FL 33136, USA

**Keywords:** Multiple Sclerosis, Relapsing-Remitting Multiple Sclerosis, Progressive Multiple Sclerosis, transglutaminase-2, blood–brain barrier disruption, perivascular lesion, endothelial inflammation, extracellular matrix

## Abstract

Multiple Sclerosis (MS) is a chronic CNS autoimmune disease characterized by immune-mediated demyelination, axon loss, and disability. Dysregulation of transglutaminase-2 (TG2) has been implicated in disease initiation and progression. Herein, TG2 expression in post-mortem human brain tissue from Relapsing Remitting MS (RRMS) or Progressive MS (PMS) individuals were examined and correlated with the presence of TG2 binding partners and effectors implicated in the processes of inflammation, scar formation, and the antagonism of repair. Tissues from Relapsing-Remitting Multiple Sclerosis (RRMS; *n* = 6), Progressive Multiple Sclerosis (PMS; *n* = 5), and non-MS control (*n* = 6) patients underwent immunohistochemistry for TG2, PLA2, COX-2, FN, CSPG, and HSPG. TG2 was strongly upregulated in active RRMS and PMS lesions, within blood vessels and the perivascular tissue of sclerotic plaques. TG2 colocalization was observed with GFAP+ astrocytes and ECM, including FN, HSPG, and CSPG, which also increased in either RRMS or PMS lesions. Although TG2 was not colocalized with inflammatory mediators COX-2 and PLA2, or the macrophage-microglia marker Iba1, its increased expression correlated with their elevation in active RRMS and PMS lesions. In summary, the correlation of strong TG2 induction in either RRMS or PMS with some of its binding partners but not others implicates potentially different roles for TG2 in disparate MS forms that may warrant further investigation.

## 1. Introduction

Multiple Sclerosis (MS) is a debilitating and chronic autoimmune neurodegenerative disease of the central nervous system (CNS) that exhibits inflammation mediated demyelination [1,2]. MS leads to diverse neurological deficits, progressive loss of sensorimotor function, and clinical disability [3,4]. The etiology underlying MS pathogenesis has been proposed to involve environmental factors, particularly exposure to the Epstein–Barr virus (EBV) and vitamin D insufficiencies as well as ultraviolet light exposure, smoking, and stress. When combined with unique genetic predispositions related to gender and ethnicity, these factors contribute to MS susceptibility [5,6].

The clinical disease course of MS can be highly variable, with four distinct clinical subtypes identified. In the clinically isolated syndrome (CIS), only a single episode of neurological symptoms resulting from inflammation or demyelination in the CNS might occur. However, most MS patients (>85%) experience an inflammation-induced, relapsing remitting (RRMS) form of the disease that exhibits acute exacerbations with one or more neurological deficits, followed by a partial or complete recovery of symptoms. Whereas RRMS occurs in the initial stage of MS, in the later stages of the disease, around 65% of patients with RRMS will convert to a non-relapsing form, with a progression of neurological symptoms and disability. Secondary progressive MS (SPMS) is associated with a degenerative condition of the neuron–axonal axis. Lastly, 10–15% of MS patients are diagnosed with primary progressive MS (PPMS), where neurological deficits continue to worsen gradually from the disease onset [7].

The immunopathophysiology of MS remains to be fully elucidated, although the dysregulation of both the innate and the adaptive immune response is involved [8,9], leading to the aberrant immune targeting of oligodendrocytes and the myelin sheath. The key non-CNS cellular components of MS pathophysiology include CD4+ Th1 and Th17 cells, CD20+ B cells, CD8+ T cells, monocytes, neutrophils, macrophages, and dendritic cells [10,11]. The interactions of these immune cells with CNS resident glia, microglia, and astrocytes are important for disease pathogenesis [8,9,12]. Acute and progressive phases of MS, however, do exhibit disparities in which immune components are involved, with peripheral immune cells predominating in early disease and resident microglia playing a greater role in progressive disease [9,12]. Further disparities exist in the dysregulation of inherent immunosuppressant control mechanisms [13,14], the integrity of the blood–brain barrier (BBB) [15], and the CNS tissue presence of autoreactive T and B lymphocytes as well as monocytes [16,17,18,19]. Acute RRMS exhibits BBB disruption, immune cell infiltration, and glial reactivity across multifocal plaques of axon demyelination that can then undergo a remission phase of repair and remyelination [20]. Conversely, progressive MS (PMS) disease occurs with compartmentalized, chronic inflammatory lesions exhibiting an intact BBB, demyelination, axon injury, and neurodegeneration that is associated with insufficient repair to reverse continued or persistent functional loss [21]. Despite the advances in identifying the cellular and molecular mechanisms underlying disparate forms of MS and the translation of novel therapeutics to reduce the occurrence, duration, and severity of disease relapses, a cure for MS remains elusive.

Transglutaminase-2 (TG2) is a calcium-dependent, protein-crosslinking enzyme [22,23,24,25,26] that is ubiquitously expressed. TG2 possesses a wide variety of activities, from acting as a GTPase and kinase to a transamidase and scaffolding protein with many substrates across various intracellular compartments, the cell surface, and within the extracellular space. These interactions are important for cell survival [27,28] and the phagocytic function of the macrophages in tissue repair [29,30,31], but can also contribute to increased inflammation [32] and cell-death [33] in neurodegenerative conditions [34], including Huntington disease (HD) [35], Alzheimer disease (AD) [36], Amyotrophic Lateral Sclerosis (ALS) [37], and Multiple Sclerosis (MS) [38,39,40,41]. Recently, our group and others identified TG2 dysregulation in cadaveric MS brain samples and in the CNS of rodents with EAE (experimental autoimmune encephalomyelitis) [38], highlighting its role in the pathogenesis of MS. In EAE, TG2 is expressed in infiltrating monocytes [41], promoting their adhesion and tissue migration into the CNS [41]. Sestito et al. [39] demonstrated that this elevated TG2 in the monocytes of MS patients correlated with the progression of the clinical course of disease in MS [39,42], suggesting that TG2 is an important therapeutic target to alter the disease course in MS. Extracellular TG2 also crosslinks with various extracellular matrix (ECM) molecules such as Fibronectin (FN) [43] and Heparin Sulfate Proteoglycan (HSPG) [44], which are known to mediate inflammation and promote tissue scarring, which is inhibitory to repair [45]. Selective deletion of TG2 in astrocytes has been demonstrated to perturb the production of the chondroitin sulfate proteoglycan (CSPG) NG2 after spinal cord injury (SCI) [46] and to improve neuronal survival when these TG2 depleted astrocytes are co-cultured with neurons in vitro following exposure to cytotoxic stimuli [47]. We recently showed elevated expression of endothelial and neuronal TG2 acutely after EAE in mice and rats and in MS brain tissue [38], where TG2 likely contributes to endothelial inflammation and the formation of sclerotic plaques as well as neuron degeneration [38] and apoptosis via cleaved caspase-3 activity [48]. Though the precise cellular role of TG2 in MS remains to be elucidated, global TG2 knockout or TG2 inhibition is associated with perturbed induction and reduced disease severity in EAE.

In the present study, we sought to examine how TG2 expression may be disparately altered in CNS sclerotic plaques from cadaveric RRMS and PMS patients. We also assessed how TG2 was spatially correlated to the localization of proinflammatory mediators, FN, CSPG, HSPG, cPLA2, and COX-2, which are known to functionally interact with, or are regulated by, TG2.

## 2. Materials and Methods

Human brain specimens: Human post-mortem brain tissue from RRMS (*n* = 6), PMS (*n* = 5), or non-MS controls (*n* = 6) were obtained from The Human Brain and Spinal Fluid Resource Center (VA West Los Angeles Healthcare Center, Los Angeles, CA, USA). The brain tissue specimens were derived from subjects between the ages of 38 and 82 years and included both genders, though a preponderance of male samples did exist, as documented in Table 1. Brain specimens were examined and the presence of typical demyelinating lesions that were consistent with MS were confirmed by a neuropathologist at The Human Brain and Spinal Fluid Resource Center. A waiver from The University of Miami IRB was obtained. The cadaveric tissue samples did not contain any of the 18 identifiers noted in the HIPPA privacy rule.

Tissue processing: Frozen tissue blocks from cadaveric human brains of non-MS (control) specimens and from the two forms of relapsing remitting and progressive MS were fixed by immersion in 4% paraformaldehyde solution (phosphate buffer, pH 7.0) for a period of 48 h at 4 °C, replacing with fresh paraformaldehyde solution at 24 h. Fixed tissues were washed twice with phosphate buffer and dehydrated in 30% sucrose solution and stored at 4 °C prior to cryosectioning. After rapidly embedding the samples in OCT compound (EMS, #62550-01), cryosectioning was performed. Coronal sections of the tissues (20 µm thickness) were cut using a cryostat and mounted on charged glass slides (TruBond 380 Adhesive Microscope Slides, IHC World, IW-T380c). Slides were then dried at room temperature for at least one hour prior to storage at −20 °C until further immunohistological processing.

Reagents: Table 2 shows the list of different primary antibodies, their source, and the dilutions that were employed in the study for immunohistochemistry.

Histology and immunocytochemistry: For visualization of tissue pathology and to identify plaques of demyelination and lesion activity, cryosectioned tissue sections were stained with Hematoxylin, Eosin and Luxol Fast Blue (LFB) [49]. The stage of the lesion was defined as active when both degraded myelin and Iba1+ immune cells were present in the vicinity of the sclerotic plaques. Inactive lesions were characterized by complete demyelination along with an absence of activated immune cells.

For immunohistochemistry, heat antigen retrieval was first performed on the sectioned tissue, as described previously [50], followed by blocking in 2% bovine serum albumin (BSA) in PBS (phosphate-buffered saline) with 0.5% Triton-X100 for an hour at room temperature. Blocked tissue was subjected to specific primary antibody treatment overnight at room temperature. Primary antibodies employed were validated against their specific target proteins in previously published studies or by the manufacturer. Sudan Black was used to block tissue autofluorescence in pathological specimens to eliminate false positive signals by CNS lipids. Antibody immunoreactivity (IR) was visualized using fluorophore conjugated secondary antibodies. Sectioned tissues with only a secondary antibody added were employed as controls to ensure that the fluorescent signal observed was specific to the primary antibodies employed.

Imaging and analysis: Image acquisition of the stained tissue was carried out using a slide scanner or a confocal laser-scanning microscope (Olympus, Fluoview FV 1000, Center Valley, PA, USA). For batch acquisition of high resolution images of the tissue stained with Hematoxylin, Eosin, and Luxol Fast Blue, the images were acquired using the brightfield batch scan function on the Olympus VS120 Fluorescence and Brightfield Slide Scanner for High-Throughput Image Acquisition. Images of immunostained tissue sections were taken using the fluorescent scan function at 20× magnification. The images were imported and processed using OlyVIA software. The fluorescent scans were performed using DAPI, FITC, TRITC, and CY5 filter cubes. The images of each fluorescent channel were exported in grayscale, pseudo-colored, and overlaid to form a multichannel composite image using Adobe Photoshop 22 (Adobe Systems Inc., San Jose, CA, USA). Immunostained images were exported in Big TIFF format and further processed by performing a 2 × 2 bin of each image in Adobe Photoshop 22 (Adobe Systems Inc., San Jose, CA, USA) for converting the files to a TIFF format for the purpose of size reduction, without altering the clarity and the resolution of the images. To maintain image consistency, all images were processed with the same exposure adjustment of +2.80 and offset adjustment of −0.03.

For acquisition of immunofluorescent images using the confocal laser scanning microscope, images were obtained from at least three randomly selected fields from the region of interest (ROI) within the stained tissue sections. Immunofluorescent intensity was measured to determine the antibody reactivity for each of the target proteins and quantified using Image J software (available online: http://imagej.nih.gov/ij/, accessed on 13 December 2019). The sharpness and the tonal range (smart sharpen, 0.9 pixels) of the Tiff files of the images were normalized using Adobe Photoshop CS2 (Adobe Systems Inc., San Jose, CA, USA). All cadaveric samples were imaged and analyzed according to HSB# ID only by an individual who was blinded to the neuropathological identification and classification of the sample as MS or control. Samples obtained from The Human Brain and Spinal Fluid Resource Center (Los Angeles, CA, USA) were randomly provided based upon number of samples requested. Note that the samples provided did contain an unusually high allocation of males for a condition that clinically shows a greater prevalence in females.

Statistical analysis: Quantitative data obtained from measurements of the immunofluorescent intensity of different staining combinations were plotted as the mean ± standard deviation of the mean (SD). Statistical analysis was performed, employing an analysis of variance (ANOVA) followed by a Tukey test for multiple comparisons between the two forms of MS and the non-MS control group using GraphPad Prism v7.0. (GraphPad Software, Inc., La Jolla, CA, USA). Differences were accepted as statistically significant at * *p* < 0.05, ** *p* < 0.01, or *** *p* < 0.0001. To investigate whether TG2 immunoreactivity levels were related to disease progression and/or the stage of the lesion in patients with the two forms of MS, linear regression analyses were performed between TG2 IR of 3 or more randomly selected lesions from each section/patient and the corresponding immunoreactivity of specific immunopathological markers or pathogenic ECM as stated in each figure. The *p*-value determined by linear regression and Pearson correlation coefficient (r) are indicated in each of the figures.

## 3. Results

**Figure 1:**
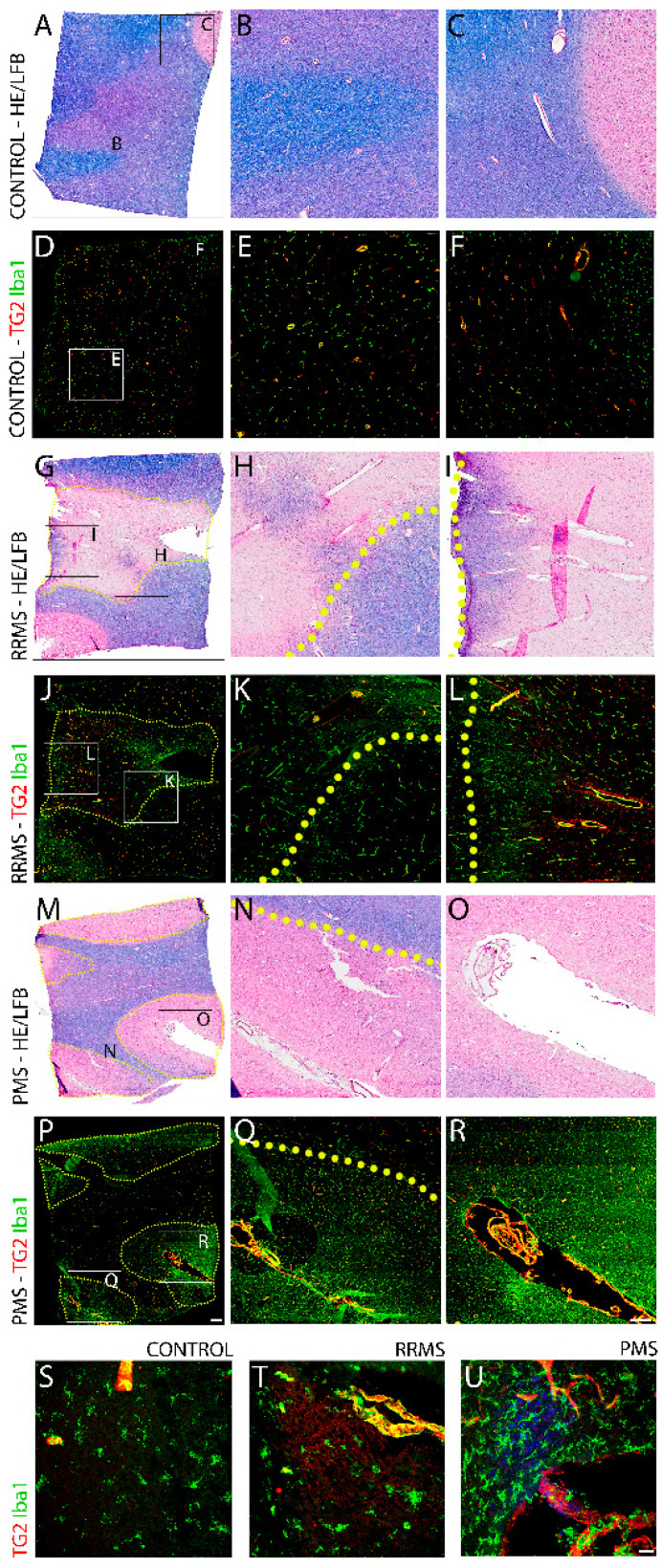
Comparative gross pathology of cortical lesions after RRMS and PMS. Brain tissue sections from control non-MS (**A**) and after RRMS (**G**), and PMS (**M**) were stained with Hematoxylin, Eosin, and Luxol Fast Blue (**A**,**G**,**M**) to delineate white matter perivascular lesions with demyelination. An adjacent tissue section from each sample was subjected to immunohistochemistry (**D**,**J**,**P**, respectively) using antibodies towards TG2 (Red) and the macrophage-microglia specific marker Iba1(Green). Scale bars = 1000 µm. Close up views of specific regions of H&E/LFB stained section (**A**,**G**,**M**) are shown in (**B**,**C**,**H**,**I**,**N**,**O**), respectively, while close up views of specific regions of Iba1 and TG2 immunostained sections of (**D**,**J**,**P**) are shown in (**E**,**F**,**K**,**L**,**Q**,**R**), respectively. Magnified views of microglia and macrophages can be seen in Iba1+/TG2 double stained brain sections from control (**S**), RRMS (**T**), and PMS (**U**) patients. The control tissue from non-MS patients shows a microglial morphology of a typical resting phenotype that is highly branched and has small cell bodies. Stained section from RRMS patients showed microglial cells with an activated phenotype surrounding active lesions with the cells having an ameboid morphology with larger cell bodies and short processes. Tissue sections from PMS patients showed highly dense regions of activated microglia within and around active lesions exhibiting shorter processes and larger cell bodies. The density of activated microglia and macrophages surrounding active lesions in tissue sections from PMS patients appeared significantly higher than that from RRMS patients.

PMS exhibited significantly higher Iba1 immunoreactivity than RRMS, whereas the strong TG2 induction in both forms was comparable. Comparative pathological assessment was conducted on cadaveric cortical brain tissue samples obtained from 17 individuals, 6 with RRMS, 5 with PMS, and 6 non-MS controls. The clinical characteristics of the three groups are presented in Table 1. Randomized imaging of at least three demyelinated plaques and ensuing histological evaluation was performed on tissue sections stained with Hematoxylin, Eosin, and Luxol Fast Blue. Profound demyelinated lesions were observed in the white matter of both RRMS (Figure 1G–I) and PMS (Figure 1M–O) specimens compared to normal appearing myelin in the non-MS controls (Figure 1A–C). The gross pathological examination revealed similar lesion sizes and morphology between both MS forms, though a significant disparity in immune cell density was identified upon additional immunostaining for the marker Iba1. Cellular immunoreactivity for Iba1 was present at demyelinated white matter lesions, with marked density in perivascular tissues surrounding inflamed, strongly TG2-positive blood vessels. The numbers of Iba1+ cells were significantly greater in PMS specimens (Figure 1P–R) than those of RRMS (Figure 1J–L), with these cells around and within the TG2 immunoreactive tissue areas and blood vessels at the sclerotic lesions. The presence of Iba1 would be indicative of either macrophage CNS tissue infiltration or activation and focal migration of reactive microglia to the lesion. The normal-appearing white matter (NAWM) and analogous tissue regions from the non-MS control exhibited only sparse Iba1 immunoreactivity (Figure 1D–F).

**Figure 2:**
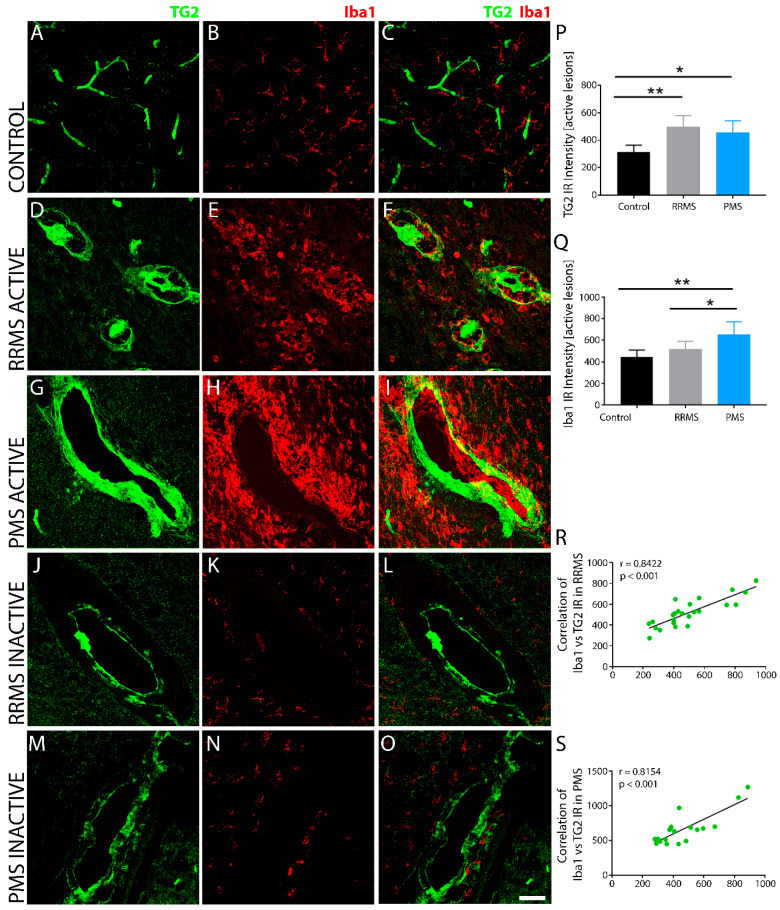
TG2 immunoreactivity is significantly upregulated in active lesions of MS brain tissue. Compared to non-MS brain tissue (**A**–**C**), which exhibited low basal levels of TG2 expression (**A**,**C**) and a sparse population of Iba1+ immune cells (**B**), TG2 expression was robustly increased in active lesions of both RRMS (**D**,**F**) and PMS (**G**,**I**). A dense cellular immunoreactivity for Iba1+ activated microglia and macrophages was also observed in and around the lesions (**E**,**H**). TG2 and Iba1-positive immune cell density were both significantly lower in inactive lesions of both RRMS (**J**–**L**) and PMS (**M**–**O**). Quantitative assessment of TG2 (**P**) and Iba1 (**Q**) immunoreactivity in active lesions of RRMS and PMS brain tissues revealed significantly increased expression levels compared to non-MS brain tissue. A positive linear correlation was observed between TG2 immunoreactivity and the density of Iba1-positive immune cells surrounding active lesions in both RRMS (**R**) and PMS (**S**). Scale bar = 40 µm. Data were quantified and expressed as value of the mean plus the standard deviation (SD). Statistical significance indicated a * *p* < 0.05; ** *p* < 0.01, determined using a 1-way analysis of variance and post hoc Tukey test. The correlation between the immunodensity of Iba1 and TG2 IR in the lesions from all the patients in each of the two forms of MS is shown in (**R**,**S**). The *p*-value determined by linear regression and Pearson correlation coefficient (r) is indicated for each of the analyses.

Quantitative evaluation of TG2 immunoreactive density within active sclerotic plaques of confocal images revealed no differences in staining between RRMS (Figure 2D,F) and PMS (Figure 2G,I) samples, although the immunofluorescence intensity was significantly greater than that of the non-MS controls (Figure 2A,C). Comparative regions in the non-MS controls had significantly less TG2 expression, with strong but restricted immunoreactivity only in the blood vessels (Figure 2A,C). In the MS specimens, TG2 in the sclerotic plaques was associated with high accumulation of Iba1+ immune cells in perivascular tissue areas (Figure 2E,F,H,I), with less pronounced Iba1 immunoreactivity in the active lesions of RRMS (Figure 2E,F) compared to the active PMS (Figure 2H,I) lesions. The extent of TG2 expression in the sclerotic plaques in both forms of MS was not uniform and the level of its expression appeared to be dependent on the stage of the lesion. Inactive lesions exhibited lower TG2 immunoreactivity than active lesions in both RRMS (Figure 2J,L) and PMS (Figure 2M,O). TG2 staining appeared patchy within blood vessels and perivascular tissues that also exhibited sparse, low intensity cellular Iba1 immunoreactivity (Figure 2K,L,N,O). Linear regression analysis of the correlation between TG2 and Iba1 immunoreactive density within active lesions of RRMS (Figure 2R) and PMS (Figure 2S) samples showed a significant, positive correlation for both forms.

**Figure 3:**
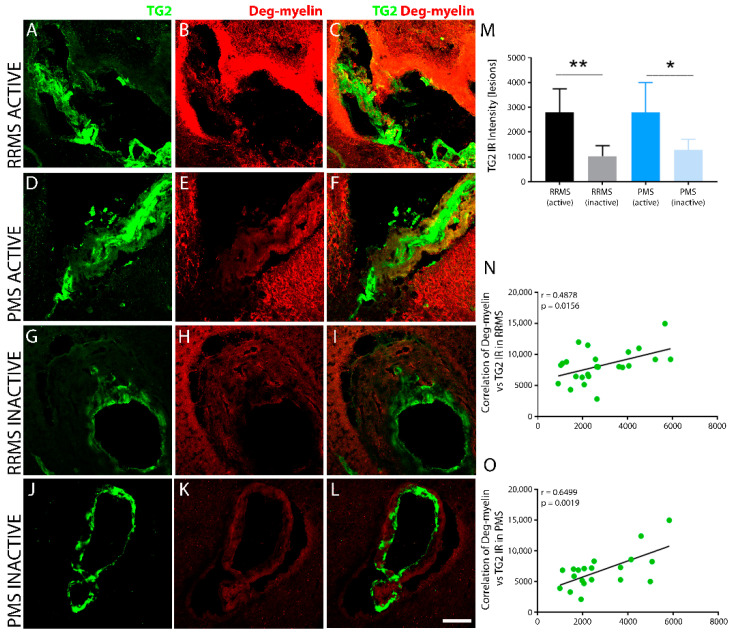
TG2 expression correlates with degraded myelin in active lesions after MS. Active lesions in brain tissues from both RRMS (**A**–**C**) and PMS (**D**–**F**) showed elevated levels of TG2 (**A**,**C** and **D**,**F**) respectively. TG2 correlated with levels of degraded myelin surrounding sclerotic plaques of both forms of MS (**B**,**C** and **E**,**F**, respectively). Inactive lesions from RRMS (**G**–**I**) and PMS (**J**–**L**) exhibited reduced levels of TG2 and degraded myelin. Quantitative assessment of TG2 levels in sclerotic plaques of both RRMS and PMS brain tissue exhibited significant differences in TG2 expression levels between active and inactive lesions (**M**). Scale bar = 50 µm. Data were quantified and expressed as value of the mean plus the standard deviation (SD). Statistical significance indicated a * *p* < 0.05; ** *p* < 0.01, determined using a 1-way analysis of variance and post hoc Tukey test. Degraded myelin immunoreactivity showed a significant, positive correlation with the extent of TG2 expression in lesioned tissue after both RRMS (**N**) and PMS (**O**). The correlation between levels of degraded myelin and TG2 IR in the lesions from all the patients in each of the two forms of MS is shown in (**N**,**O**). The *p*-value determined by linear regression and Pearson correlation coefficient (r) is indicated for each of the analyses.

The lesion levels of degraded myelin correlate with TG2 immunoreactivity in both RRMS and PMS. Degraded myelin was identified in tissue sections using an antibody targeted against the immunogen consisting of YGSLPQKSQRSQDENPVV MBP69-86 synthetic peptide, which recognizes degraded MBP [51]. Pronounced degraded myelin was found within sclerotic lesions of both RRMS (Figure 3A–C) and PMS (Figure 3D–F) samples, particularly within perivascular tissues. Inactive lesions displayed much lower levels of degraded myelin immunoreactivity (Figure 3M) for both RRMS (Figure 3G–I) and PMS (Figure 3J–L). Linear regression analysis of the correlation between TG2 and degraded myelin immunoreactive density within active lesions of RRMS (Figure 3N) and PMS (Figure 3O) samples showed a significant, positive correlation for both forms of MS.

**Figure 4:**
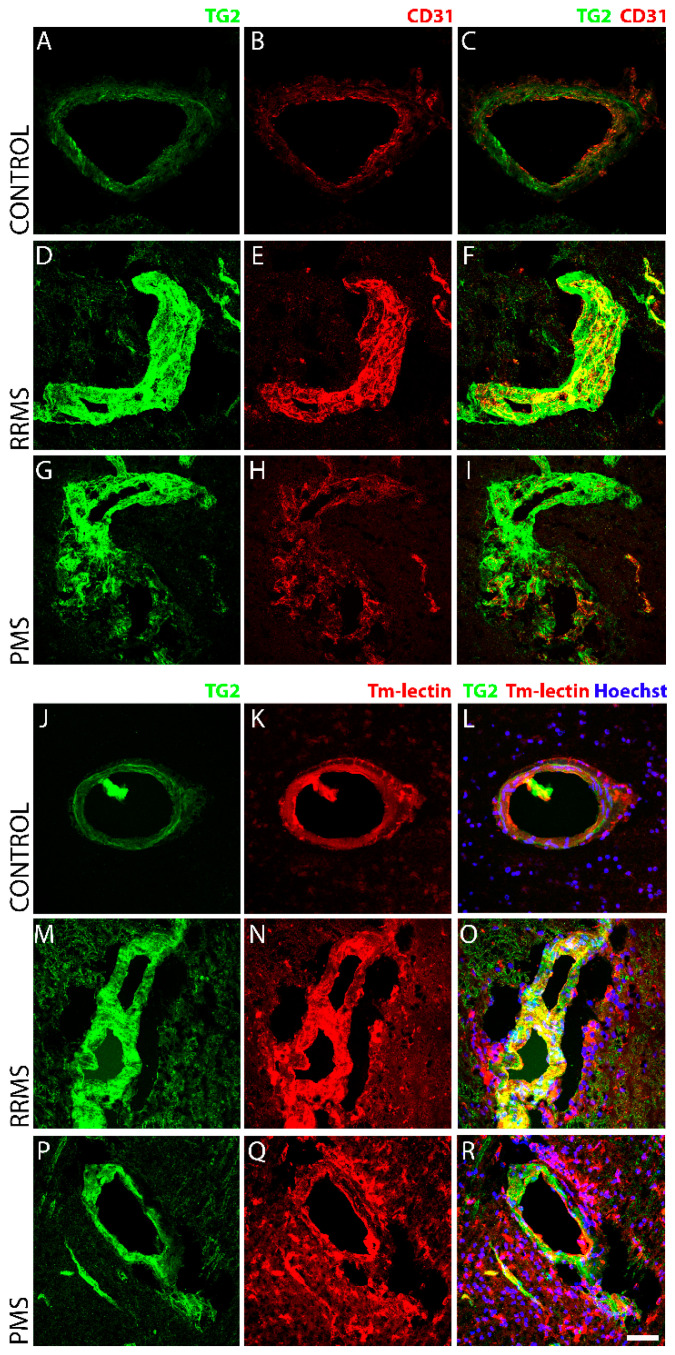
TG2 immunoreactivity is robustly upregulated in endothelial cells within active lesions of MS brain tissue. Compared to non-MS brain tissue (Top panel; **A**–**C** and Bottom panel; **J**–**L**), which showed low levels of basal TG2 expression (**A**,**C**,**J**,**L**) in the endothelial cells labelled with CD31 (Top panel, **B**,**E**,**H**) or tomato lectin (Bottom panel, **K**,**N**,**Q**), TG2 expression was robustly increased in the active lesions of both forms of MS; RRMS (Top panel **D**,**F** and Bottom panel **M**,**O**) and PMS (Top panel, **G**,**I** and Bottom panel **P**,**R**). Scale bar = 50 µm.

**Figure 5:**
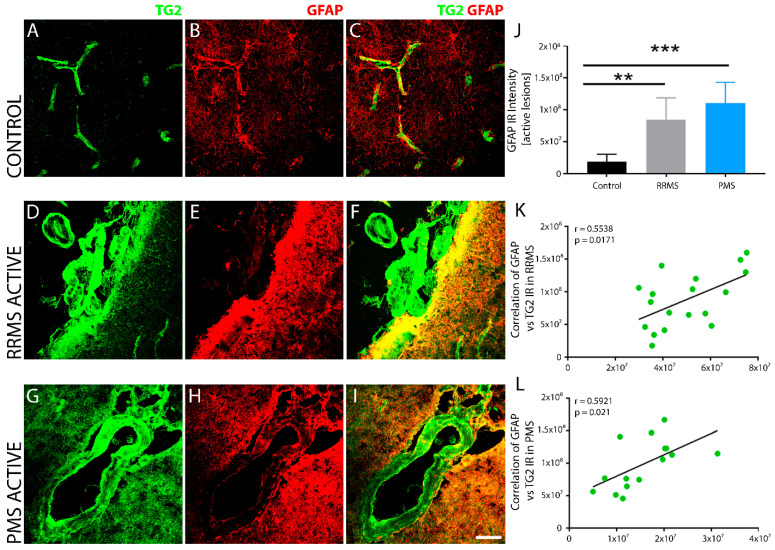
TG2 expressing sclerotic lesions are surrounded by reactive astrocytes. Compared to non-MS brain tissue (**A**–**C**), sclerotic lesions with elevated levels of TG2 showed an increase in reactive astrocytic marker glial fibrillary acidic protein (GFAP, red) surrounding the plaques in brain tissue after both RRMS (**D**–**F**) and PMS (**G**–**I**). The edge of the sclerotic plaques showed overlapping of GFAP and TG2 (green) immunoreactivity. Scale bar represents 50 µm. Data were quantified and expressed as value of the mean plus the standard deviation (SD). Statistical significance is indicated by a ** *p* < 0.001; *** *p* < 0.0001, determined using a 1-way analysis of variance and post hoc Tukey test. Quantitative measurement of total fluorescence intensity indicated a significant increase in GFAP immunoreactivity in both RRMS (*n* = 6) and PMS (*n* = 5) sclerotic plaques (**J**) compared to non-MS brain tissue (*n* = 6). GFAP immunoreactivity in lesioned tissue showed a significant positive correlation with TG2 expression in active lesions of both RRMS (**K**) and PMS (**L**). The correlation between GFAP^+^ expression and TG2 IR in lesions from all patients in each of the two forms of MS is shown in (**K**,**L**). The *p*-value determined by linear regression and Pearson correlation coefficient (r) is indicated for each of the analyses.

Strong, perivascular TG2 immunoreactivity colocalized with endothelial cells (Figure 4) and GFAP-positive astrocytes (Figure 5) in sclerotic lesions of RRMS and PMS. TG2 levels were robustly increased in the disrupted microvasculatures associated with active lesions only in both forms of MS. In blood vessels probed with CD31 (Figure 4B,E,H) or tomato lectin (Figure 4K,N,Q) (*Lycopersicon esculentum*), characteristic markers of endothelial cells, TG2 IR (Figure 4C,F,I and L,O,R respectively) was significantly enhanced in MS lesions (Figure 4D–I) compared to the controls (Figure 4A–C,J–L), while TG2 IR was not apparent in inactive or old lesions (*data not shown*). Additionally, dense GFAP immunoreactivity was found to be localized to perivascular tissue in active lesions in RRMS (Figure 5E,F) and PMS (Figure 5H,I), and was significantly increased compared to the non-MS brain tissue control (Figure 5B,C), as measured quantitatively by fluorescent intensity (Figure 5J). TG2 staining showed robust induction and colocalization with GFAP in both RRMS (Figure 5F) and PMS (Figure 5I), implicating an astrocyte contribution to TG2 upregulation in MS. The elevated expression of TG2 in astrocytes was particularly evident in perivascular regions surrounding active lesions in both forms of MS. Linear regression analysis of the correlation between TG2 and GFAP density within active lesions of RRMS (Figure 5K) and PMS (Figure 5L) showed a significant, positive correlation for both forms of MS.

**Figure 6:**
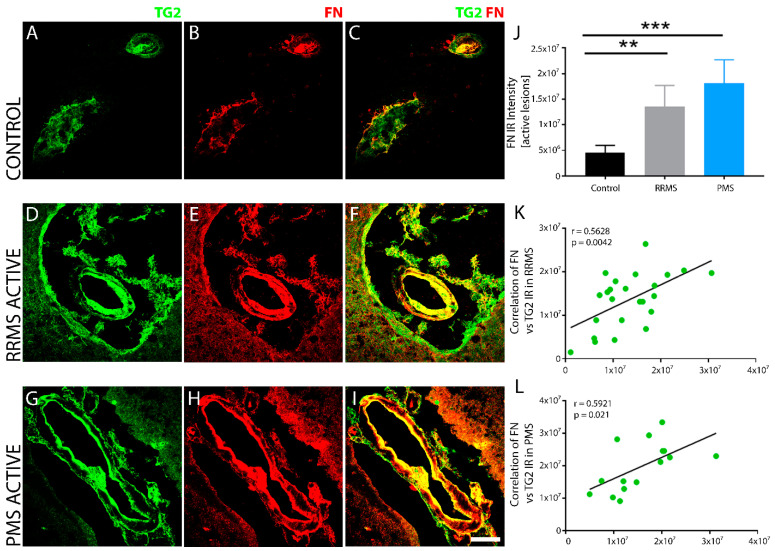
Comparative assessment of TG2 and FN co-expression in normal and MS brain tissues. The expression levels of FN and TG2 in sclerotic plaques of brain tissue after RRMS (*n* = 6) and PMS (*n* = 5) were analyzed and compared to non-MS brain tissue (*n* = 6) using double fluorescent immunohistochemistry. TG2 (green) and FN (red) colocalized (yellow) around blood vessels. Representative images of active lesions from demyelinated regions of tissue samples from the two forms of MS were acquired and compared to non-MS tissue samples (**A**–**C**). Elevated levels of FN immunoreactivity overlapped in regions that exhibited increased TG2 expression, which was more abundant in the active lesion core after both RRMS (**D**–**F**) and PMS (**G**–**I**). Baseline expression levels of FN in non-MS brain tissue were significantly lower (**J**). Scale bar = 50 µm. Data were quantified and expressed as value of the mean plus the standard deviation (SD). Statistical significance is indicated by a ** *p* < 0.001; *** *p* < 0.0001, determined using a 1-way analysis of variance and post hoc Tukey test. FN expression showed a significant positive correlation to the extent of TG2 IR in active lesions after both RRMS (**K**) and PMS (**L**). The correlation between FN expression and TG2 IR in the lesions from all the patients in each of the two forms of MS is shown in (**K**,**L**). The *p*-value determined by linear regression and Pearson correlation coefficient (r) is indicated for each of the analyses.

TG2 immunoreactivity exhibits strong overlap with FN within MS lesions of RRMS and PMS. Compared to non-MS control tissue (Figure 6B,C), FN was strongly up regulated in both RRMS (Figure 6E,F) and PMS (Figure 6H,I), as measured quantitatively by fluorescent intensity (Figure 6J). Colocalization of FN was pronounced with TG2, both within the blood vessels as well as in the surrounding perivascular tissue of RRMS (Figure 6D,F) and PMS (Figure 6G,I) lesions. No differences in FN expression or in its colocalization with TG2 were observed between MS forms. Linear regression analysis of the correlation between TG2 and FN density within active lesions of RRMS (Figure 6K) and PMS (Figure 6L) samples showed a significant, positive correlation for both forms of MS.

**Figure 7:**
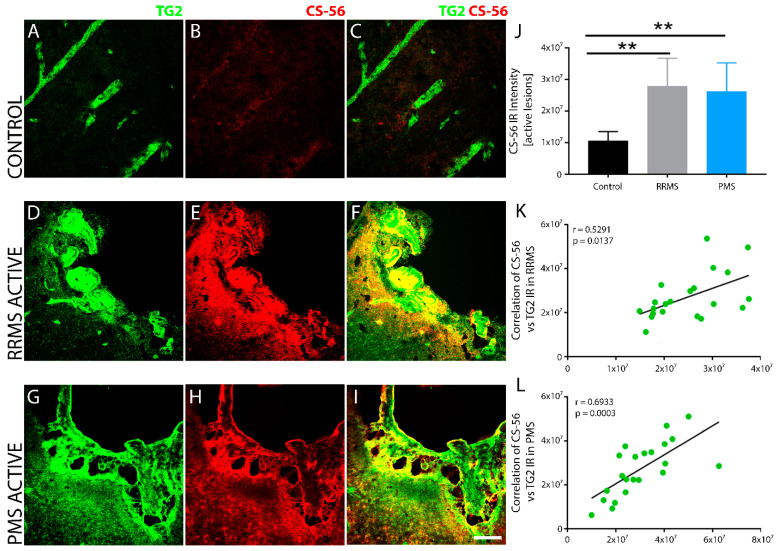
CSPG levels were upregulated with increased TG2 IR within active sclerotic lesions of both RRMS and PMS brain tissues. TG2 immunofluorescence in control, non-MS brain tissue exhibited restricted basal expression (**A**), which was largely found within blood vessels and overlapped with very low levels of CSPG immunoreactivity (CS56 antibody, **B**,**C**). Conversely, there was robust and overlapping accumulation of TG2 and CSPG, both within the core and the edge of sclerotic plaques in brain tissue from both RRMS (**D**–**F**) and PMS (**G**–**I**). High-magnification image scale bar represents 50 µm. Quantitative measurement of total fluorescence intensity of CS-56 immunoreactivity indicated a significant increase in the levels of CSPG in both RRMS (*n* = 6) and PMS (*n* = 5) sclerotic plaques (**J**) compared to the low expression levels measured in non-MS brain tissue (*n* = 6). No significant difference was observed in CSPG immunoreactivity within the active sclerotic lesions between RRMS and PMS. Statistical significance is indicated by a ** *p* < 0.01; determined using a 1-way analysis of variance and post hoc Tukey test. Error bars are mean ± SD. CSPG expression showed a significant positive correlation with TG2 IR in active lesions after both RRMS (**K**) and PMS (**L**). The correlation between the expression levels of CS-56 and TG2 IR in the lesions from all the patients in each of the two forms of MS is shown in (**K**,**L**). The *p*-value determined by linear regression and Pearson correlation coefficient (r) is indicated for each of the analyses.

Perivascular TG2 colocalizes with CSPGs in active brain lesions after MS. Robust and overlapping TG2 and chondroitin sulfate proteoglycan (CSPG), an axon growth inhibitory extracellular matrix, were observed both within the core and the edge of sclerotic plaques of RRMS (Figure 7D–F) and PMS (Figure 7G–I) specimens compared to a low basal level expression of both in non-MS control tissue (Figure 7A,B). Quantitative measurements of fluorescence intensity showed a marked increase in CSPG levels in both forms of MS, with no significant difference between them (Figure 7J). Linear regression analysis of the correlation between TG2 and CSPG density within active lesions of RRMS (Figure 7K) and PMS (Figure 7L) samples showed a significant, positive correlation for both forms of MS.

**Figure 8:**
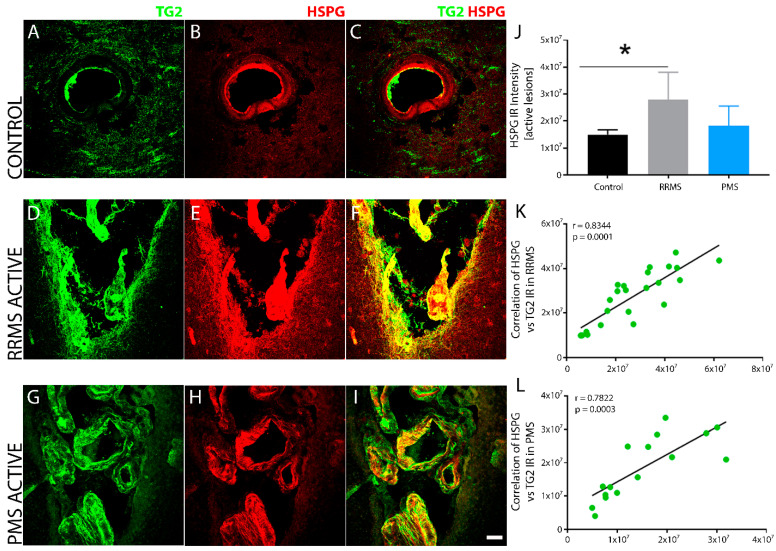
TG2 and HSPG immunoreactivity overlap in sclerotic lesions after RRMS. In contrast to non-MS control brain tissue, which exhibited lower expression levels of TG2 and HSPG in the basal lamina of the blood vessels (**A**–**C**), sclerotic lesions after RRMS (**D**–**F**) show pronounced and overlapping reactivity of HSPG and TG2 immunofluorescence both in the center and the edge of the sclerotic lesions. The extent of HSPG expression in lesions after PMS was not as robustly increased (**G**–**I**) compared to the RRMS lesions and was mostly limited to the lesion center. Quantitative assessment of HSPG immunoreactivity from active lesions of the two forms of MS indicated significant upregulation in the levels of HSPG only after RRMS and not PMS compared to non-MS controls (**J**). Scale bar = 30 µm. Data were quantified and expressed as value of the mean plus the standard deviation (SD). Statistical significance indicated a * *p* < 0.05, determined using a 1-way analysis of variance and post hoc Tukey test. The extent of HSPG expression was proportional to the lesion associated TG2 levels and showed a significant positive correlation to the intensity of TG2 IR in sclerotic lesions after both RRMS (**L**) and CPMS (**K**). The correlation between the levels of HSPG and TG2 in the lesions from all the patients in each of the two forms of MS is shown in (**K**,**L**). The *p*-value determined by linear regression and Pearson correlation coefficient (r) is indicated for each of the analyses.

Increased HSPG immunoreactivity localizes with TG2 in RRMS lesions. Compared to low basal levels of Heparan Sulfate Proteoglycans (HSPG) in non-MS control brain tissue, its immunoreactivity was significantly increased (Figure 8J) in blood vessels and perivascular tissue after RRMS (Figure 8E,F) compared to the lesions in PMS (Figure 8H,I). HSPG colocalized with TG2 after RRMS (Figure 8F) both in the lesion center as well as around the lesion edge compared to the lesions after PMS (Figure 8I), which showed increased HSPG colocalization with TG2 largely in the lesion center while the non-MS control tissue (Figure 8C) only exhibited low levels of HSPG-TG2 colocalization that was limited to the basal lamina around the blood vessels. Linear regression analysis of the correlation between TG2 and HSPG density within active lesions of RRMS (Figure 8K) and PMS (Figure 8L) samples showed a significant, positive correlation for both forms of MS.

**Figure 9:**
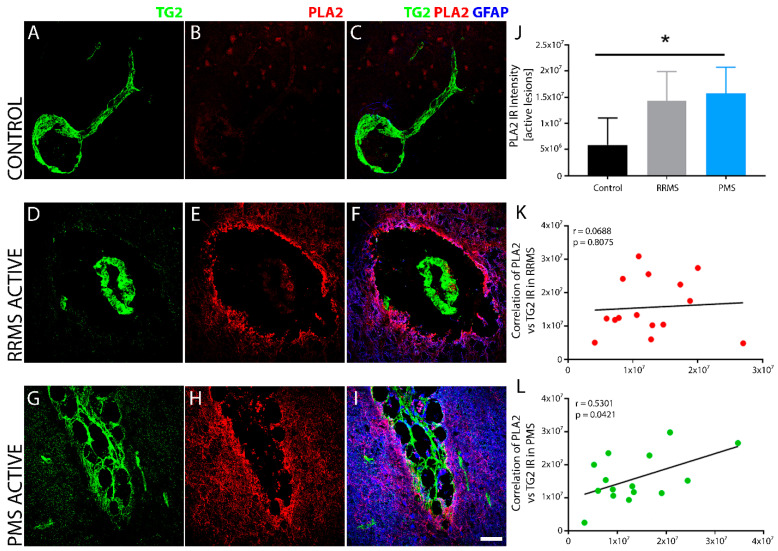
Lesions show increased phospho-PLA2^Ser505^ immunoreactivity in brain tissue with TG2 after PMS. pPLA2^Ser505^ levels were elevated with an increase in the expression of TG2 in active lesions after PMS. Most of the PLA2 immunoreactivity was found in the lesion edge, while being absent in the lesion core. The non-MS control brain tissue (**A**–**C**) did not show pPLA2^Ser50^ IR (**B**,**C**) compared to brain tissue after RRMS (**D**–**F**), which showed only modest increase in the levels of pPLA2^Ser50^ (**E**,**F**) compared to the control tissue. Sclerotic lesions after PMS (**G**–**I**) exhibited a robust increase in pPLA2^Ser50^ (**H**,**I**) expression. Quantitative assessment of pPLA2^Ser50^ immunoreactivity indicated significant upregulation in the levels of pPLA2^Ser50^ only after PMS (**J**). Scale bar = 50 µm. Data were quantified and expressed as value of the mean plus the standard deviation (SD). Statistical significance indicated a * *p* < 0.05, determined using a 1-way analysis of variance and post hoc Tukey test. pPLA2^Ser50^ expression showed a significant positive correlation with TG2 IR in active lesions only after PMS (**L**) and not after RRMS (**K**). The correlation between the expression levels of pPLA2^Ser50^ and TG2 IR in the lesions from all the patients in each of the two forms of MS is shown in (**K**,**L**). The *p*-value determined by linear regression and Pearson correlation coefficient (r) is indicated for each of the analyses.

*PMS, but not RRMS, exhibited a significant increase in inflammatory phospholipase A2, which correlated with TG2 expression.* Compared to non-MS controls (Figure 9A–C), increased levels of the proinflammatory mediator cytosolic-phospho-PLA2^Ser505^ was detected concurrently with elevated TG2 IR in sclerotic plaques of PMS (Figure 9G–I), but not RRMS (Figure 9D–F), brain tissue. Quantitative analysis of phospho-PLA2 IR revealed a significant increase in PMS lesions compared to non-MS controls (Figure 9J). Linear regression analysis of the correlation between TG2 and PLA2^Ser505^ immunoreactivity within active lesions of PMS (Figure 9L), but not RRMS (Figure 9K), samples showed a significant, positive correlation.

**Figure 10:**
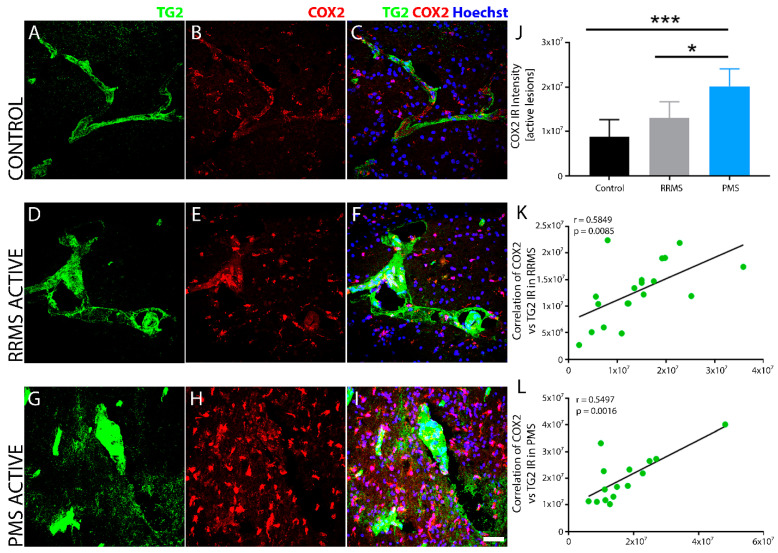
MS lesions show increased COX-2 immunoreactivity with TG2 in brain tissue after MS. Inducible COX-2 (red) levels were elevated in sclerotic lesions associated with TG2 (green) after MS. The non-MS control brain tissue (**A**–**C**) exhibited low, basal levels of COX-2 IR (**B**,**C**) compared to affected regions of brain tissue after RRMS (**D**–**F**), which showed a modest increase in COX-2 that was proportional to the level of the cellular density around the active lesion (**E**,**F**). Sclerotic lesions in PMS (**G**–**I**) exhibited a robust COX-2 expression (**H**,**I**) that was similarly localized but much higher in density (**H**). Quantitative assessment of COX-2 IR indicated significant upregulation only after PMS (**J**). Scale bar = 40 µm. Data were quantified and expressed as value of the mean plus the standard deviation (SD). Statistical significance indicated a * *p* < 0.05, *** *p* < 0.0001 determined using a 1-way analysis of variance and post hoc Tukey test. COX-2 expression showed a significant positive correlation to the extent of TG2 IR in active lesions after both RRMS (**K**) and PMS (**L**). The correlation between the expression levels of COX2 and TG2 IR in the lesions from all the patients in each of the two forms of MS is shown in (**K**,**L**). The *p*-value determined by linear regression and Pearson correlation coefficient (r) is indicated for each of the analyses.

Sclerotic plaques of RRMS and PMS exhibit elevated COX-2 with increased TG2. Cyclooxygenase-2 *(*COX-2) immunoreactivity was robustly increased in perivascular tissues of PMS lesions (Figure 10H,I) and to a lesser extent in RRMS (Figure 10E,F) compared to the non-MS brain tissue control (Figure 10B,C), as measured quantitatively by fluorescent intensity (Figure 10J). COX-2 immunoreactivity was cellular and likely associated with macrophages and microglia. TG2 staining also showed robust induction in lesions that correlated, but did not colocalize, with COX-2 in both RRMS (Figure 10D–F) and PMS (Figure 10G–I). Linear regression analysis of the correlation between TG2 and COX-2 density within the active lesions of RRMS (Figure 10K) and PMS (Figure 10L) samples showed a significant, positive correlation for both forms of MS.

## 4. Discussion

Relapsing remitting and progressive forms of MS share the pathological appearance of perivascular, demyelinating lesions within the CNS but exhibit significant differences in vascular, immunological, and neurodegenerative features. Herein we compared the expression of the multifunctional enzyme TG2 in post-mortem MS brain tissues from RRMS and PMS individuals and correlated TG2 changes to the expression of specific markers of vascular inflammation, glial, and immune cell reactivity, and scar-related ECM deposition. TG2 showed strong induction in both RRMS and PMS within the endothelial cells and intraluminal structures of the microvasculature, as well as extracellularly in perivascular tissue regions and reactive glia, particularly astrocytes. The expression of TG2 in macrophages and microglia, however, was limited in both RRMS and PMS. The increased TG2 expression in both forms of MS correlated with the prominent markers of the MS pathology examined, except for pPLA2^Ser50^ in RRMS. The present study confirms the putative pathogenic role of TG2 in MS across distinct forms of the disease.

Active lesions exhibited strong TG2 expression and were associated with the CNS accumulation of reactive microglia and macrophages in both forms of MS. The density of these immune cells was more pronounced in PMS compared to RRMS. Active lesions also showed the concurrent induction of other inflammatory mediators, including an activated form of cytosolic p-PLA2^Ser505^ and inducible COX-2, as well as scar-related ECMs such as FN, CSPG, and HSPG. These ECM molecules displayed significant colocalization with TG2, both within the architecture of the blood vessels and the inflamed perivascular CNS tissue, and within hypertrophied astrocytes surrounding the lesions. These proinflammatory molecules have been previously reported to be potential binding partners of TG2 [22,44,52,53] and provide functional interactions that mediate the intercellular signaling associated with the promotion of neuroinflammation and demyelination.

Under normal physiological conditions, enzymes of the PLA2 superfamily are involved in phospholipid metabolism; however, an increased level of cytosolic PLA2 in the CNS has been shown to induce inflammation-mediated demyelination and is a pathophysiological marker of MS [54,55]. TG2 has been reported to interact with PLA2 and is involved in the post-translational modification of this enzyme to enhance its catalytic activity. Increased PLA2 activity increases the mobilization of arachidonic acid (AA) from the membrane phospholipids [52], which is then catalyzed by the enzyme COX-2 to release eicosanoids such as prostaglandins and leukotrienes, which are known proinflammatory lipid mediators. Although low levels of COX-2 are observed constitutively in the normal CNS, where it is associated with synaptic and neurovascular functions [56], its expression is significantly increased in the presence of an inflammatory stimuli [57]. Prostaglandins (PGs) that have been demonstrated to drive an inflammatory response in various neurodegenerative conditions [58], including MS [59], are generated by inducible COX-2. Previous work has reported that a marked increase in COX-2 occurs during the relapse phase of chronic EAE [59], where it is associated with activated macrophages and microglia surrounding the sclerotic lesions [60]. Similarly, robust cellular COX-2 was expressed in the perivascular CNS tissue of PMS lesions that exhibited strong TG2, but was significantly less prevalent in RRMS lesions. The interaction of TG2–PLA2–COX-2 appears therefore to be one of the key signaling mechanisms involved in inflammation and the MS pathophysiology of the progressive disease form. Studies with cytosolic PLA2-deficient mice have shown resistance to EAE induction [55]. Collectively, these findings indicate that the functional coupling between TG2 and cPLA2 could be a critical inducer of MS pathology.

ECM produced by reactive glia have also been identified as important constituents of MS pathology [61]. TG2 within the microvasculature at the perivascular lesion sites colocalizes extracellularly with FN [38,62], where it fulfills a functional role in disease propagation as an immune cell adhesive scaffold. TG2 has a high binding affinity towards FN and cross links FN to confer increased stability and rigidity during the formation of the matrix [63]. Under normal physiological conditions, the presence of FN is limited only to blood vessels [64]. FN, however, is transiently upregulated during tissue injury and binds other ECM or regulates cell migration and proliferation through its interaction with integrin receptors. In MS lesions, FN has been shown to be a major impediment to remyelination repair by inhibiting oligodendrocyte progenitor cell (OPC) maturation [64,65]. The upregulation of FN appears to be a characteristic response in MS lesions and functions to promote leukocyte migration and impair remyelination, irrespective of the type and stage of MS disease. The vascular TG2–FN scaffold sequesters circulating immune cells at the site of BBB breakdown and allows them to then extravasate into surrounding CNS tissue to potentiate damage, demyelination, axon degeneration, and neuronal loss.

Although the active lesions of RRMS and PMS both exhibited strong vascular and perivascular TG2 immunoreactivity, there was a difference in the upregulation of its potential binding partners between MS forms, with a robust presence of HSPG2 in RRMS but not PMS and alternatively strong phospho-PLA2^Ser505^ in PMS but not as much in RRMS. A linear correlation between the expression levels of TG2 and the activation of cPLA2 was statistically significant only after PMS and not in the RRMS lesions. The functional significance of this disparity in the disease course is unknown but the availability of TG2 and PLA2 knockout mice would allow for examination of whether the removal of one or the other would have a concurrent effect. Genetic ablation of TG2 [66,67] or PLA2 [55] is associated with the antagonism of EAE induction.

Abnormal tissue remodeling and ECM production in the regions of demyelination of the brain and spinal cord after MS have been widely reported in MS [68,69,70]. ECM deposition of regeneration-inhibitory proteoglycans at the edge of the perivascular lesions has been implicated in lesion expansion, scar formation, abortive axon regeneration, and the inhibition of remyelination repair [61,70]. In the examination of RRMS and PMS brain tissues at lesion sites, increased levels of both FN and CSPG colocalize with strong TG2. CSPG deposition around the lesion edge after MS, in both RRMS and PMS, was observed in the current study and has been also reported elsewhere [71]. CSPG is a growth inhibitory substrate produced primarily by reactive astrocytes and other glia surrounding the lesion [72] and is known to perturb OPC migration and thus antagonize remyelination. CSPGs also prevent OPC maturation and thus in MS are a major impediment for repair. Here we have shown that TG2 and CSPG colocalize within the perivascular tissue in both RRMS and PMS. Although there is no evidence of a direct protein–protein interaction between TG2 and CSPGs, recent work has reported a diminution of CSPG production, when TG2 is selectively knocked out in astrocytes following spinal cord injury [46,73] and that such a manipulation leads to improved functional recovery.

Another ECM that is upregulated with MS is perlecan or HSPG2, a subtype of HSPG, which functions as a reservoir for growth factors, including vascular endothelial growth factor (VEGF), transforming growth factor (TGFβ), and platelet-derived growth factor (PDGF), which are critical in intercellular interactions [74]. Perlecan also plays an important role in maintaining the stability and functional integrity of the BBB [75] and has been implicated as a pro-regenerative substrate for damaged axons [76]. In contrast to these supportive actions, HSPGs have also been associated with the inhibition of myelination [77] that would antagonize repair in MS. We found that perlecan colocalizes with TG2 in active lesions after RRMS but not CPMS, suggesting a differential function of TG2–HSPG interactions across MS disease forms. In addition, endothelial TG2 has been reported to interact with HSPGs and prevent heparin sulfate VEGF-induced angiogenesis [78]. Abnormal angiogenesis is a known pathological component of human MS [79] and is associated with the severity of the disease. Whether TG2–perlecan interactions in RRMS are beneficial, contributing to the repair of the BBB or remyelination repair through the release of VEGF or PDGF during the remission phase of the disease, requires further investigation. Similarly, a lack of TG2–HSPG interaction in the active lesions of PMS may be associated with loss of endogenous repair and disease progression, a phenomenon that would need to be substantiated.

In summary, the present work reveals that in different forms of MS, RRMS and PMS, the differential availability of inflammatory and ECM binding partners of TG2 at the sites of perivascular lesions and strong TG2 induction may confer very different pathological roles for TG2 in disease remission or progression. Further examination of these interactions in animal models of disease and the use of genetic knockout strategies will shed light on whether these differences play important roles in the pathological course of either MS disease form or whether such interactions could be a therapeutically targetable direction for peptide-based approaches. Indeed, the peptide inhibition of TG2–FN interactions has been demonstrated to alter TG2-dependent cell-ECM adhesion and spreading [80].

## Figures and Tables

**Table 1:** Source of tissue specimens from control and multiple sclerosis patients employed in the study.

HSB# Patient ID	Age (years)	Gender	Microscopic Description of Lesion from the Neuropathological Report	Post-Mortem Interval (hours)	Clinical Diagnosis
3422	62	Male	Section of periventricular white matter showed demyelination characterized by a variably decreased axonal density (80–100%) and complete demyelination. There was severe associated gliosis and oligodendrocyte loss but no associated macrophage activity or perivascular lymphocytic cuffing. Presence of chronic MS plaque formation.	11.75	MS/RRMS
3891	53	Male	Section of periventricular white matter showed plaque formation with up to 100% axonal loss and 100% demyelination, near complete loss of oligodendrocytes, and associated gliosis was prominent along with scattered evidence of macrophage activity and perivascular lymphocytic cuffing Presence of chronic/active multiple sclerosis plaque formation and active multiple sclerosis plaque formation.	25.3	MS/CPMS, depression, post-traumatic stress disorder, degenerative spine disease
5053	67	Male	Section of periventricular white matter showed plaque formation with up to 100% axonal loss and 100% demyelination There was near complete oligodendrocyte loss and prominent associated gliosis. There was no evidence of macrophage activity or perivascular lymphocytic cuffing Evidence of chronic multiple sclerosis plaque formation.	24.9	MS/secondary progressive MS, migraine, history of stroke, transient ischemic attack (TIA), Chronic Obstructive Pulmonary Disease (COPD), pneumonia, aspiration, meningitis, ataxia, chronic UTI
5095	60	Male	Section of periventricular white matter showed irregular but fairly well-defined area of extensive myelin loss and relative preservation of axons with scant perivascular mononuclear infiltrates. There was associated gliosis at the periphery of the lesion.	8.1	MS, RRMS, sclerosis, heart attack
5102	60	Female	Section of periventricular white matter showed fairly well-defined area of demyelination and relative preservation of axons with scattered macrophages.	14.7	MS, RRMS, depression, celiac disease, headache. Neurogenic bladder, chronic UTI, osteoporosis, insomnia
5123	79	Female	Section of periventricular white matter displayed somewhat ill-defined areas of myelin pallor with relative axonal preservation.	24.8	MS/RRMS, congestive heart failure, neuralgia, trigeminal, asthma
5139	81	Male	Section of periventricular white matter showed myelin loss with relative axonal preservation consistent with chronic inactive plaque of multiple sclerosis. Evidence of demyelinating lesions consistent with clinical history of multiple sclerosis.	36.8	MS/RRMS, dysphagia, paraplegic, neurogenic bladder, diabetes type 11, hypertension, hyperlipidemia, myocardial infarction (Ml), atherosclerosis
5154	63	Male	Section of periventricular white matter showed irregular but fairly well-defined area of extensive myelin loss and relative preservation of axons with rare perivascular mononuclear infiltrates. There was associated mild gliosis at the periphery of the lesion.	12.6	MS/RRMS, optic neuritis
5160	82	Male	Sections of periventricular white matter showed plaque formation with up to 100% axonal loss and 100% demyelination with near-complete oligodendrocyte loss and associated gliosis. There was no evidence of macrophage activity or perivascular lymphocytic cuffing. Chronic multiple sclerosis plaque formation.	9.8	MS/RRMS, stroke/CVA, pneumonia, aspiration, MRSA (methicillin-resistant Staphylococcus aureus), chronic UTI
5170	52	Female	Sections from the lateral angle of the lateral ventricle showed an old demyelination plaque surrounded by hypercellular white matter. Presence of old demyelination plaque surrounded by hypercellular white matter.	25.0	MS, CPMS, optic neuritis, depression
5270	38	Female	Old demyelination plaques were seen in the sections of posterior cingulate gyrus and pons. Presence of old demyelination plaques.	17.5	MS, CPMS, asthma, depression, pain, paraplegic, ataxia (cerebellar)
4307	84	Male	Normal Appearing White Matter (NAWM)	11.8	CA, stomach, renal failure, acute, COPD (control, non-MS)
4308	70	Male	NAWM	11.8	Coronary heart disease, leukemia, diabetes type I, myocardial infarction (control, non-MS)
4294	80	Male	NAWM	19.2	CA, pancreas, hypertension (control, non-MS)
4631	59	Male	NAWM	20.2	COPD, pulmonary emphysema, congestive heart failure, tobacco abuse, atrial fibrillation, hypertension (control, non-MS)
5072	83	Male	NAWM	19.5	COPD, seizure disorder (clinical only), atrial fibrillation (control, non-MS)
4615	49	Male	NAWM	15	CA, colon with metastasis to liver, depression (control, non-MS)

*The demographic data were obtained from The Human Brain and Spinal Fluid Resource Center (Los Angeles, CA).*

**Table 2:** Primary antibodies used for immunohistochemistry.

Primary Antibody	Manufacturer	Catalog Number	Antibody Host	Dilution Used
TGM2 pAb (CUB 7402)	Thermo Fisher	MA5-12739	Mouse	1:100
Rb pAb to Transglutaminase-2	Abcam	ab421	Rabbit	1:100
Anti IBA1, Rabbit (for ICC)	Wako/Fuji	019-19741	Rabbit	1:1000
Chicken Polyclonal to IBA1	Encor	CPCA-IBA1	Chicken	1:1000
Goat pAb to IBA1	Abcam	ab5076	Goat	1:500
Myelin Basic Protein	Encor	CPCA-MBP	Chicken	1:2500
Degraded Myelin Basic Protein	Millipore	AB5864	Rabbit	1:1000
Glial Fibrillary Acidic Protein	Dako	Z0334	Rabbit	1:500
Anti-Glial Fibrillary Acidic Protein	Millipore	AB5541	Chicken	1:250
Anti-Fibronectin antibody	Sigma	F3648	Rabbit	1:200
Monoclonal Anti-Chondroitin Sulfate (Clone CS-56)	Sigma	C8035 (SAB4200696)	Mouse	1:200
Anti-Heparan Sulfate Proteoglycan, (Perlecan), clone 5D7-2E4	Sigma	MABT 12	Mouse	1:200
Anti-phospho-c-PLA2 (pSer505)	Sigma	SAB4503812	Rabbit	1:100
Anti-COX-2/Cyclooxygenase 2	Abcam	ab15191	Rabbit	1:100

## Data Availability

The study did not report any data.
